# Fast quantification of extracellular vesicles levels in early breast cancer patients by Single Molecule Detection Array (SiMoA)

**DOI:** 10.1007/s10549-021-06474-3

**Published:** 2021-12-21

**Authors:** Carlo Morasso, Alessandra Ricciardi, Daisy Sproviero, Marta Truffi, Sara Albasini, Francesca Piccotti, Federico Sottotetti, Ludovica Mollica, Cristina Cereda, Luca Sorrentino, Fabio Corsi

**Affiliations:** 1 Laboratory of Nanomedicine, Istituti Clinici Scientifici Maugeri IRCCS, Pavia, Italy; 2 Genomic and Post-Genomic Center, IRCCS Mondino Foundation, Pavia, Italy; 3 Breast Unit, Istituti Clinici Scientifici Maugeri IRCCS, Pavia, Italy; 4 Medical Oncology Unit, Istituti Clinici Scientifici Maugeri IRCCS, Pavia, Italy; 5 Colorectal Surgery Unit, Fondazione IRCCS Istituto Nazionale dei Tumori di Milano, Milan, Italy; 6 Dipartimento di Scienze Biomediche e Cliniche “L. Sacco”, Università di Milano, Via G.B. Grassi, 74, 20157 Milan, Italy

**Keywords:** Breast cancer, Extracellular vesicles, Biomarker, SiMoA, Immunoassay, Tetraspanins

## Abstract

**Purpose:**

Preliminary reports suggest that extracellular vesicles (EVs) might be a promising biomarker for breast cancer (BC). However, the quantification of plasmatic levels of EVs is a complex task. To overcome these limitations, we developed a new, fast, and easy to use assay for the quantification of EVs directly in plasma based on the use of Single-Molecule Array (SiMoA).

**Methods:**

By using SiMoA to identify CD9+/CD63+ EVs, we analyzed plasma samples of 181 subjects (95 BC and 86 healthy controls, HC). A calibration curve, made of a serial dilution of lyophilized standards from human plasma, was used in each run to ensure the obtainment of quantitative results from the assay. In a subgroup of patients, EVs concentrations were estimated in plasma before and after 30 days from cancer surgery. Additional information on the size of EVs were also acquired using a Nanosight system to obtain a clearer understanding of the mechanism underlying the releases of EVs associated with the presence of cancer.

**Results:**

The measured levels of EVs resulted significantly higher in BC patients (median values 1179.1 ng/µl *vs* 613.0 ng/µl, *p* < 0.0001). ROC curve was used to define the optimal cut-off level of the test at 1034.5 ng/µl with an AUC of 0.75 [95% CI 0.68–0.82]. EVs plasmatic concentrations significantly decreased after cancer surgery compared to baseline values (*p* = 0.014). No correlation was found between EVs concentration and clinical features of BC.

**Conclusion:**

SiMoA assay allows plasmatic EVs levels detection directly without any prior processing. EVs levels are significantly higher in BC patients and significantly decreases after cancer surgery.

**Supplementary Information:**

The online version contains supplementary material available at 10.1007/s10549-021-06474-3.

## Introduction

Despite the progress made in understanding the tumorigenesis process and treatment of breast cancer (BC), it remains the most commonly diagnosed cancer and a major burden for women [1]. The identification of new easily measurable biomarkers for the diagnosis and the monitoring of the disease thus remain a priority. Recent studies have shown how extracellular vesicles (EVs) have a promising use as cancer biomarkers in general, and more in particular in BC [2–6]. EVs are lipid bilayer membrane vesicles nanometer-sized (50–150 nm) secreted from many cell types. The biochemical composition of EVs depends on the cell of origin and reflects its functional state. In effect, they are made by a complex mixture of proteins, lipids, nucleic acids, and micro-RNAs. Initially considered as garbage bags of metabolism and cell damage, extracellular vesicles have been more recently recognized as part of the intercellular communication system [7] and they are identified as a potential tool for liquid biopsy in cancer [8]. When EVs come into contact with their target cells they can transfer their cargo information modifying the target cellular expression producing different physiological and pathological responses.

The use of EVs as a liquid biopsy approach for the diagnosis, classification, and prognosis of BC attracted the interest of multiple groups [9]. In the literature, several EVs associated proteins have been suggested as possible specific markers for detecting EVs released by cancer cells [10]. For example, EDIL3 [11] and Claudin7 [12] have been presented as possible makers of early breast cancer. Other studies, including smaller cohorts of subjects, also pointed out the possible use of EGRF [13], EpCAM [14], GPC-1 [15], CD82 [16], and Mucin-1 [17] present on the surface of cancer released EVs as possible diagnostic tools. More recently, the development of analytical technologies able to simultaneously measure multiple markers also opened the possibility of using panels of proteins to identify an EVs fingerprint characteristic of BC [18, 19].

CD63 and CD9 are tetraspanins considered universal markers of EVs and are commonly used to characterize EVs extracted from biofluids. There is a particular interest in studying their expression in BC as CD63 in EVs seems to correlate with the tumor metastatic propensity inversely [20], and pieces of evidence attest that the concentration of tetraspanins CD9, CD63 is significantly higher in EVs originating from cancer cells than those derived from normal mammary cells [21]. However, more importantly, as CD63 and CD9 are also expressed on the surface of EVs released from different cell types, the study of their plasmatic levels could allow obtaining information on the organism's response to the presence of cancer.

Despite this growing interest in the field, the clinical data on the potential of EVs as an effective biomarker for cancer, particularly for BC, are still not fully convincing.

While some authors tend to consider the levels of circulating EVs as a biomarker [22], other reports seem to downplay EVs’ potential [23]. The contradictory results present in the literature are primarily due to the fact that the quantification of EVs through traditional techniques remains a challenging and complex task. In most cases, it is impossible to quantify EVs directly in the biological fluids, but it is necessary to purify them with complex, inefficient, expensive, and time-consuming techniques [24–27].

Starting from these premises, we considered the possibility that quantifying EVs using the associated tetraspanins CD63 and CD9 with an approach that does not require any pre-purification step or complex sample preparation protocol might be a way to quantify EVs levels in a more reliable and fast way.

The Single Molecule Array (SiMoA) technology is a new ultrasensitive digital ELISA immunoassay that can quantify very low concentrations of protein biomarkers present in the biological fluids [28, 29]. A recent study demonstrated how by using the SiMoA technology it was possible to study EVs plasma levels in pancreatic cancer patients without any purification process, thus reducing the time requested for the analysis and offering the possibility to analyze a greater number of samples [30]. The obtained data however were not quantitative and did not properly describe the profile of EVs distribution in different subjects.

The primary objective of the present study was to assess the difference in EVs plasma levels between early breast cancer patients (stage I–II) and healthy controls (HC) through an approach not requiring the prior isolation of EVs from plasma. To this scope, we developed a SiMoA assay for the ultra-sensitive detection of EVs based on the use of anti CD9 and anti CD63 antibodies.

## Materials and methods

### Patient selection

From February 2020 to May 2021, all consecutive patients referred to the EUSOMA-accredited Breast Unit at Istituti Clinici Scientifici Maugeri (Pavia, Italy) were screened for eligibility. Inclusion criteria were: invasive carcinoma of the breast candidate for surgical resection; age > 18; pT1-2 and pN0-N1a cancers. Exclusion criteria were the presence of distant metastases, synchronous presence of a different tumor, or indication for neoadjuvant chemotherapy. A control group made of sex- and age-matched healthy volunteers, not affected by cancer or chronic diseases was also enrolled. The study was conducted following the International Conference on Harmonization [ICH] Good Clinical Practice [GCP] guidelines. The Ethical Committee of ICS Maugeri authorized the study as protocol 2490/2020. All patients who agreed to participate signed a specific informed consent prior to the inclusion in the study.

### Blood collection

Samples were measured at the laboratory of Nanomedicine and Molecular Imaging Laboratory at Istituti Clinici Scientifici Maugeri Pavia (Italy). Blood samples were collected in EDTA-coated tubes. The blood samples were then centrifuged at 2000×*g* for 10 min at 24 °C. Plasma samples were collected and centrifuged a second time with a mini centrifuge at 2500×*g* for 10 min at 4 °C. The plasma was then collected stored at − 80 °C until use.

### Materials

Antibodies and EVs standard from human plasma were obtained from HansaBioMed (Tallin, Estonia). The anti-CD63 mouse monoclonal unconjugated (Product Code: HMB-CD63-100 HansaBioMed) was used as the capture antibody. Anti-CD9 mouse monoclonal biotin-conjugated (Product Code: HBM-CD9B-100 HansaBioMed) was selected as the detection antibody. To determine the calibration curve, sequential dilutions of EVs standards extracted from plasma of healthy donors using a proprietary protocol based on a combination of SEC and tangential flow filtration (Product Code: HBM-PEP-100/2 HansaBioMed) were used. The certificate of analysis and a basic characterization of the standard provide by the supplier has been included as supplementary information. The set-up of the EVs detection assay was based on the use of a SiMoA homebrew assay starter kit (Product Code: 101351 Quanterix).

### Extracellular vesicles detection assay

Following the manufacturer’s guideline, the paramagnetic carboxylated beads (Quanterix) were activated with 1-ethyl-3-(3-dimethylaminopropyl) carbodiimide hydrochloride (EDC) 10 mM (Thermo Fisher Scientific) and then they were conjugated with the anti CD63 capture antibody with a working concentration of 0.1 mg/mL and stored in the bead diluent buffer. A two-step configuration protocol was optimized for the assay. For each sample, a few microliters of plasma (3–8 µL) were diluted with an appropriate volume of sample detector diluent buffer to the optimal concentration to a final volume of 100 µL. Next, 20 µL of anti-CD9 detection antibody at a working concentration of 0.2 µg/mL, and 25 µL of the previously conjugated magnetic beads were incubated for 20 min. The obtained immunocomplex was magnetically collected, washed, and re-suspended in 100 µL of Streptavidin-ß-galactosidase (SGB) solution included in the homebrew assay starter kit. After a second washing step, samples and Resorufin ß-D-galactopyranoside (RGP) substrates were loaded into an SR-X instrument (Quanterix) for the analysis that is conduct autonomously by the system.

### Nanosight analysis

For the measure of the dimension, EVs must be isolated by ultracentrifugation before the analysis. The isolation step followed a previously published protocol here briefly reported [31]. Platelet-free plasma was centrifuged at 20,000×*g* for 1 h with Centrifuge 5427 R (Eppendorf, Italy). The obtained pellet contains EVs. The pellet was washed with 0.22 µm filtered PBS and centrifuged a second time 1 h at 20,000×*g*. The resulting pellet was then processed for EVs analysis. Nanoparticle-tracking analysis (NTA) was performed using an NS300 instrument (NanoSight, Amesbury, UK). For a more accurate detection, samples were diluted with filtered PBS to the optimal concentration (10^8^–10^9^ particles/ml). After dilution, 1 mL of diluted sample was loaded in the machine and read at a rate of about 30 frames/sec. Particle movement videos were recorded 3 times per test and dimension analyzed by the NTA software (version 2.2, NanoSight). The results of NTA analysis are here presented as geometric means of the dimension obtained by the accumulation of three independent acquisitions from the same sample.

### Statistical analysis

Given the exploratory nature of the study and the lack of previous data regarding this specific issue, a sample size was not calculated a priori. For continuous quantitative variables, we first applied the Shapiro–Wilk and Kolmogorov Smirnov tests in each group to verify the normal distribution of data; we considered data as normal only if both tests accepted the null hypothesis of normal distribution. Then we performed a parametric [t-test] when the data were normal distributed or a non-parametric Mann–Whitney or a Kruskal–Wallis test to compare variables with a non-normal distribution. The confidence level was set at 95%. In order to verify correlation between two different continuous variables, we evaluated Spearman if data were not normal or Pearson correlation if data were normal, the choice was based on variables’ distribution. A ROC with relative AUC was designed to assess accuracy, sensitivity and specificity. An internal validation of accuracy was performed with bootstrap method: the original patient population was re-sampled 500 times and the optimism index (the mean of differences between AUC on bootstrap sample and AUC on original sample) was calculated. Optimism is the amount by which the AUC (or “the apparent prediction accuracy”) overestimates the true prediction accuracy of the model. Then, the corrected AUC after bootstrap was reported. Data analysis was performed using OriginLab, SAS software [v. 9.4 SAS Institute Inc., Cary, USA] and R software [v. 3.5.1, R Foundation, Vienna].

## Results

### Characteristics of study population

In total, 181 women were included in the study, namely 86 healthy subjects and 95 patients with BC. The mean age of the healthy control population was 61.0 [range: 26–88]. For the BC group the mean age was 64.0 [range: 39–92]. Healthy and BC groups were similar in terms of age (*p* = 0.15) and body mass index (*p* = 0.33). Table 1 reports the main characteristics of the two groups of subjects and a description of BC studied.

**Table 1:** Characteristics of breast cancer patients vs. healthy controls

	Healthy controls (*n* = 86)	Breast cancer (*n* = 95)	*p* value
Age (years)	61 (26–88)	64 (39–92)	0.15*
BMI (Kg/m^2^)	24.8 (15.6–36.9)	23.9 (14.3–37.8)	0.33*
pT			
1	–	76 (80%)	–
2	–	19 (20%)	–
pN			
0	–	75 (78.9%)	–
1	–	20 (21.1%)	–
Grading			
1	–	7 (7.4%)	–
2	–	64 (67.4%)	–
3	–	24 (25.2%)	–
Molecular subtype			
Luminal A	–	73 (76.8%)	–
Luminal B	–	14 (14.7%)	–
Triple negative	–	8 (8.4%)	–
Median plasma concentrations of EV (ng/µL)	613.0 [IQR: 765.2]	1779.1 [IQR: 4072.2]	< 0.0001*
(Range)	30.0–6862.0	72.1–22,805.9	

*p-value from Mann–Whitney Test

### Performance of the SiMoA assay

**Fig. 1:**
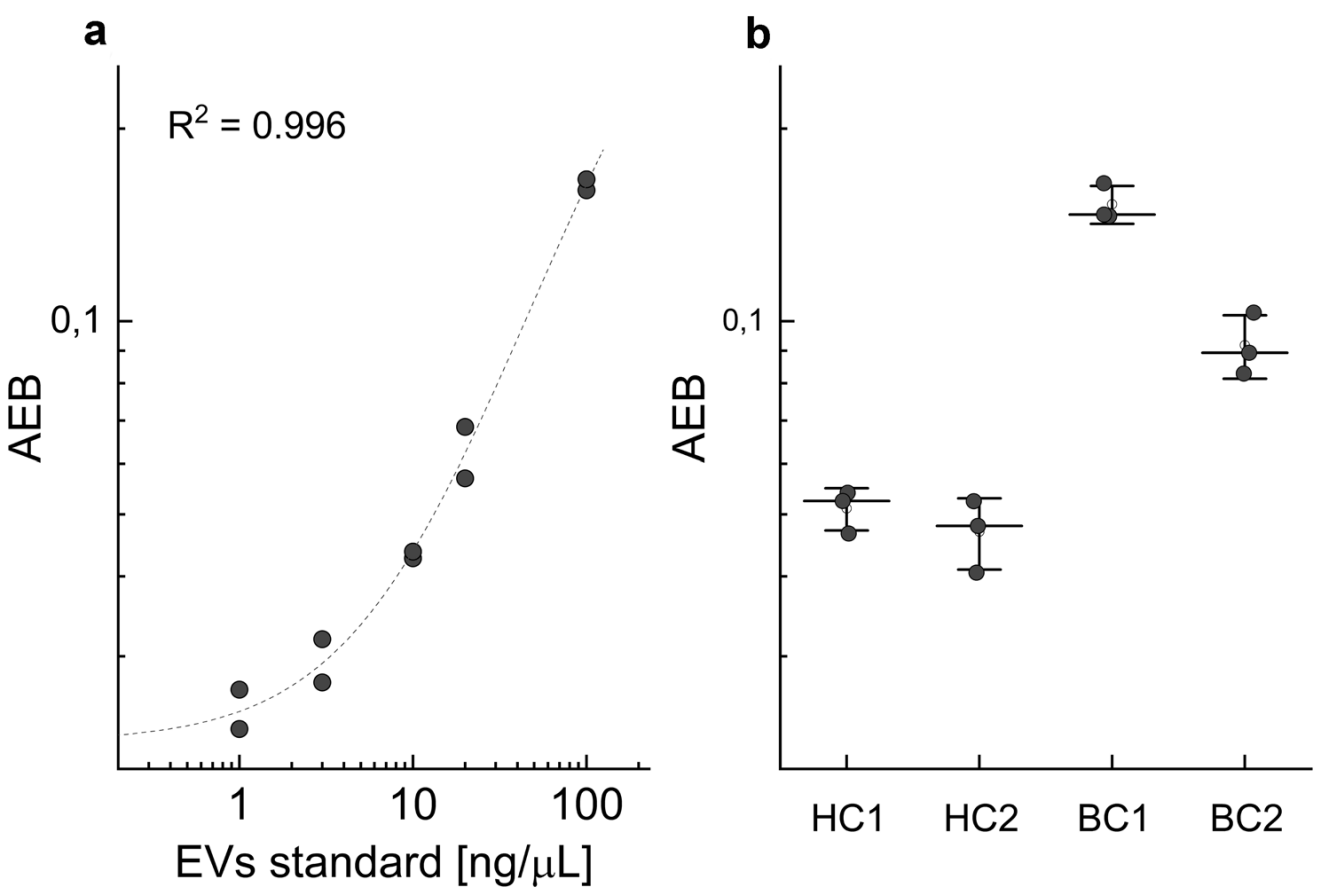
Calibration curve of the SiMoA assay. Each concentration is measured in duplicate. Data are fitted with a four-parameter logistic model (**a**). Results from the analysis of four subjects included in the study measured in triplicate. Each dot represents one measure; the central line is the mean value for each sample (**b**)

SiMoA is a new kind of analytical assay that allows the detection of very low amounts of EVs (close to a single EV) directly from the biofluid of interest. The developed assay was based on the use of magnetic beads with a diameter of approximately 2 microns conjugated to an anti CD63 antibody (capture Ab). Capture beads were mixed with a few microliters of plasma (4 µL) diluted to the final volume of 100 µL in an appropriate buffer. Afterwards an anti-CD9 biotinylated antibody (detection Ab) is added, and the mixture is incubated for 20 min. If the sample results being too concentrated for the quantification, a further dilution might be done, resulting in a reduction of the volume of plasma to be used for the analysis. In a second step, a streptavidin conjugated beta-galactosidase is added to the mixture and form a complex that is then separated from the supernatant by a magnet and washed several times. Finally, beads are loaded by the instrument in a microfluidic chip that assure that each magnetic bead is isolated in a femtoliter-sized well with the appropriate substrate. The calibration curve obtained using the SiMoA SR-X instrument was analyzed by a four-parameter logistic regression [32] which allowed to estimate a limit of quantification (LOQ—calculated as 10 standard deviations above background) in the range of 2–3 ng/µL depending on the specific plate and batch of beads used (Fig. 1a). The coefficient of variation of the assay, calculated from the triplicate analysis of the same sample, remains typically below 10% (Fig. 1b).

Each plasma sample is made in contact with about 5 × 10^5^ beads in the assay. However, not all the beads are loaded by the system in a microwell. Besides, since the SiMoA technology relies on the AEB (Average Enzyme Activity) to quantify the amount of target present in a sample, thus only beaded microwells positive to the enzyme activity are considered in the final analysis. In the presented assay, this number is typically in the range of 10^3^ and represents the number of beads effectively used.

### Plasma levels of EVs

**Fig. 2:**
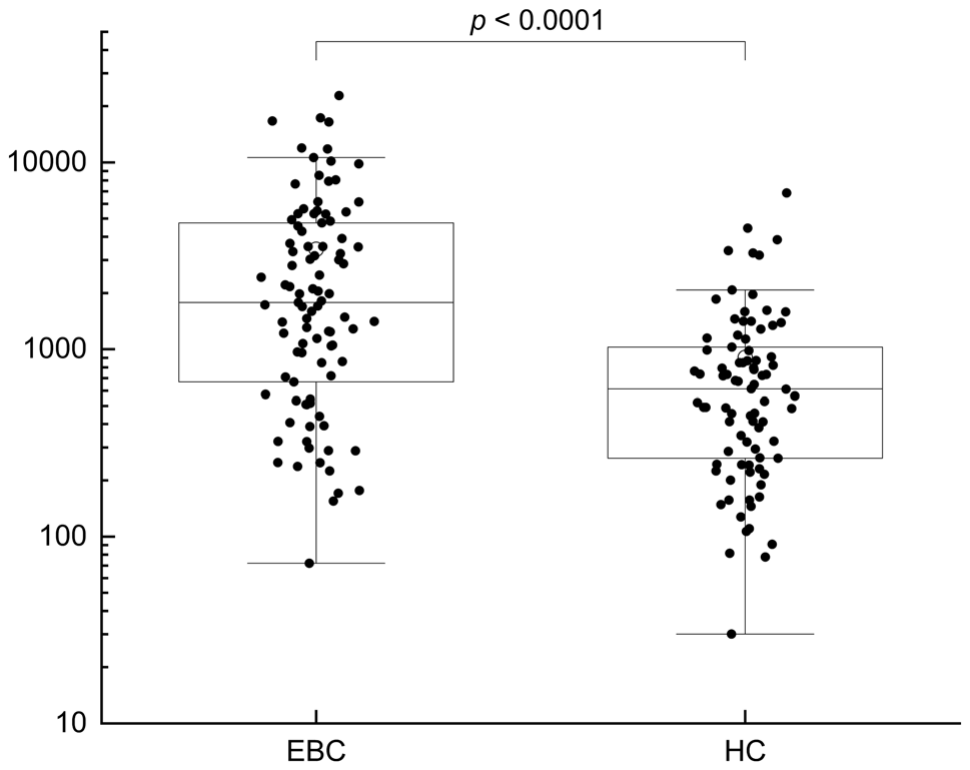
Plasmatic EVs level measured in BC (*n* = 95) and HC (*n* = 86). Data are shown as box and whisker plots. Each data point represents an individual subject analysed. Each box represents the area between the 25th and 75th percentiles [interquartile range, IQR]. Lines inside the boxes represent the median values. White dots represent the mean value for each class. Whiskers extend to the lowest and highest values within 1.5 times the IQR from the box (a)

**Fig. 3:**
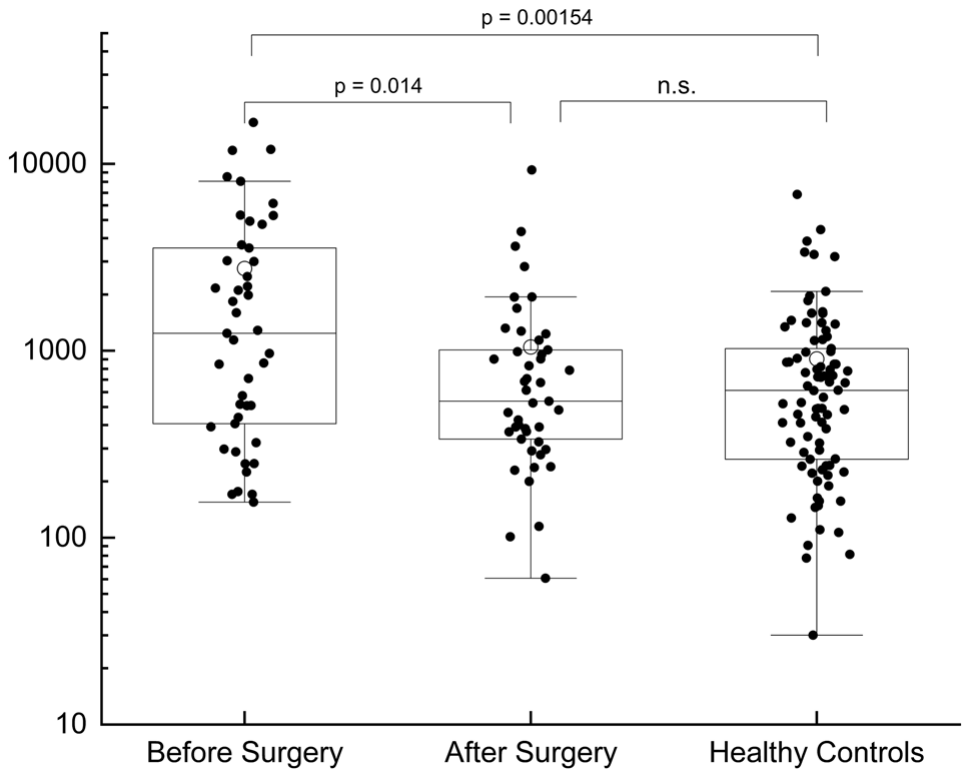
Plasma levels of EVs in BC patients before and after surgery (*n* = 45). From the results obtained, it is possible to see that one month after the operation there is a statistically significant decrease in the levels of EVs that becomes equal to the one observed on HC. Each data point represents an individual subject analysed. Each box represents the area between the 25th and 75th percentiles [interquartile range, IQR]. Lines inside the boxes represent the median values. White dots represent the mean value for each class. Whiskers extend to the lowest and highest values within 1.5 times the IQR from the box

Plasma levels of EVs were measured using the developed SiMoA assay in the whole population included in the study. EVs plasma levels resulted significantly higher in BC subjects if compared to the HC’s EVs plasma concentration. Indeed, median value for HC subjects was 613.0 ng/µL [Range: 30.0–6862.0] and for BC subjects was 1779.1 ng/µL [Range: 72.1–22805.9], p < 0.0001 (Table 1, Fig. 2). Age and BMI were not associated with EVs level for both BC and HC subjects (Supplementary Table S1). To check the accuracy of EVs in BC, ROC curve was used to define the optimal cut-off level of the test at 1034.5 ng/µl. The apparent sample out AUC was 0.754 [CI 0.68–0.82]. Bootstrapped AUC was 0.749. Sensitivity of 68% and a specificity of 75% (PPV: 75.6% and NPV 68.4%). The ROC curve is presented as Supplementary Fig. S1. In a subgroup of 45 patients, plasma samples were collected both at diagnosis and 30 days after surgery to assess possible variations in EVs concentrations. In this subgroup of patients the median EVs level was 1238.7 ng/µL [Range: 154.9–16,678.9] before surgery *vs* 537.2 ng/µL [Range: 60.6–9276.9] after breast cancer resection (*p* = 0.014), as reported in Fig. 3.

### Correlations with the clinical characteristics of BC

**Fig. 4:**
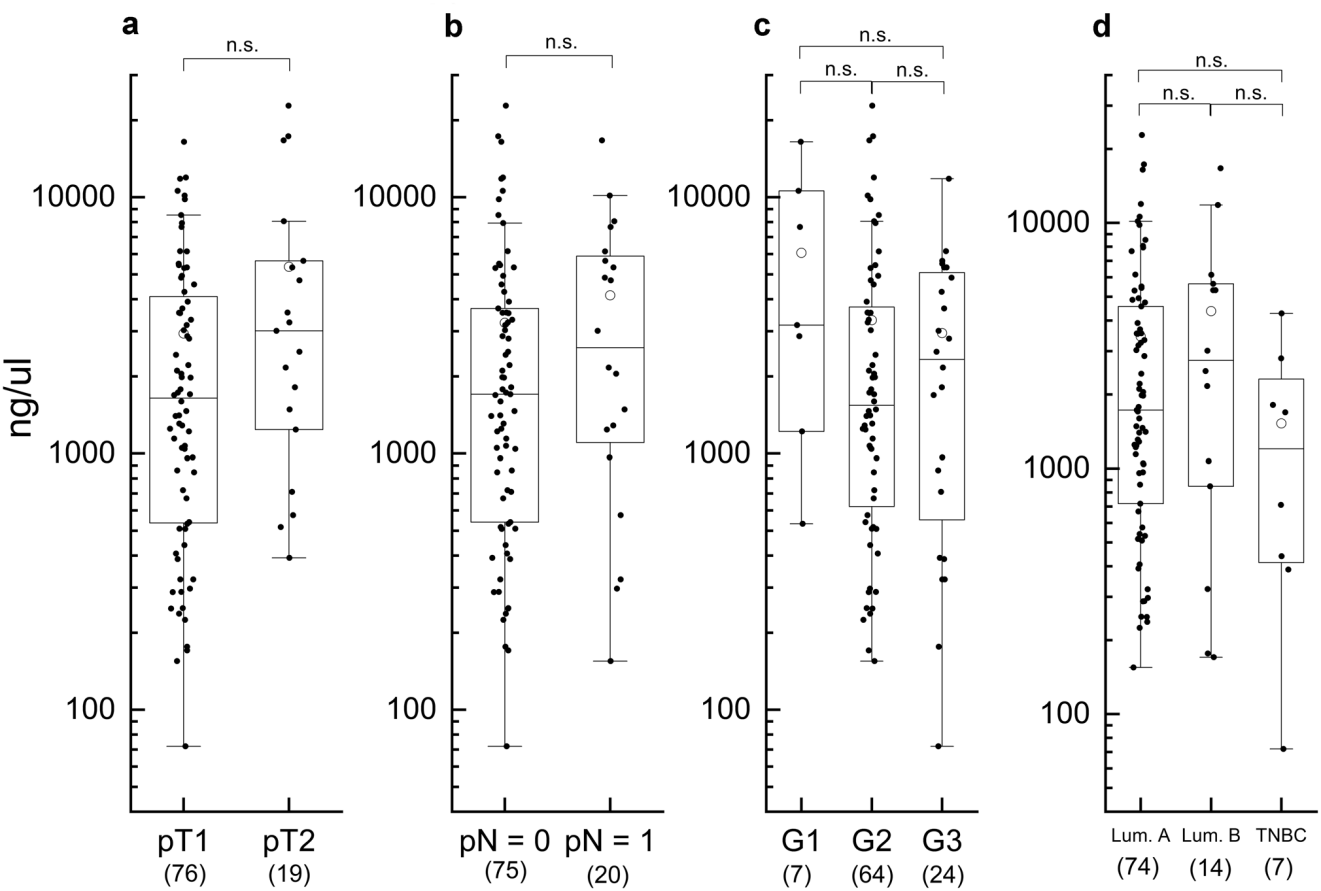
Plasmatic EVs levels observed in BC patients with different tumor size (**a**), nodal involvement (**b**), grading (**c**), and molecular subtype (**d**). Data are shown as box and whisker plots. Each data point represents an individual subject analysed. Each box represents the area between the 25th and 75th percentiles [interquartile range, IQR]. Lines inside the boxes represent the median values. White dots represent the mean value for each class. Whiskers extend to the lowest and highest values within 1.5 times the IQR from the box

Since our results indicated that plasmatic EVs levels were higher in BC patients before surgery, we investigated the possible association between EVs concentration with main clinical features. EVs concentration was not found to be correlated with pT stage (*p* = 0.07), axillary status (*p* = 0.23), grading (*p* = 0.15) and molecular subtype (*p* = 0.53), as reported in Fig. 4 (Supplementary Table S2).

### Dimension of EVs

On a subgroup of samples (*n* = 16), we explored the relationship between the EVs levels measured by the SiMoA assay in the untreated plasma and the size of the EVs as measured by Nanosight after their extraction. EVs were extracted by differential centrifugation and re-suspended in PBS. To dissect a possible influence of the disease on the size we studied samples with different characteristics: namely we analyzed BC patients with high levels of EV; BC patients with low levels of EV (below the cut-off level); and HC subjects. The data obtained did not show any correlation between the plasmatic levels of EVs and their size (Supplementary Fig. S2).

## Discussion

Liquid biopsy in an early cancer setting allows a rapid and longitudinal re-assessment by minimally invasive blood sampling, to monitor the disease and to personalize the therapeutic approach. An increasing interest in liquid biopsy has been reported in recent years, mainly due to two advantages. First, it could avoid invasive bioptic procedures, often uncomfortable or even unfeasible depending on metastatic site. Secondly, it might anticipate the presence of metastatic disease earlier than standard clinical practice, where distant lesions are revealed only by conventional imaging.

In the present study, a significantly higher concentration of circulating EVs was detected in a cohort of 95 BC patients, compared to healthy subjects (*p* < 0.0001). Interestingly, included patients were affected by early stage BC since 80% of them had T1 cancer and the great majority (78.9%) of cases were node-negative. The presence of circulating tumor-derived EVs has been frequently detected in solid tumors with distant metastases, but paradoxically monitoring of disease at a molecular level is much less relevant in this setting. Detecting tumor-derived EVs would be of major importance in early-stage BC, because variations in EVs concentration could offer fine monitoring of distant metastatic disease or cancer response to therapies, provided that EVs are detectable at baseline, allowing a prompt tailoring of therapeutic approaches. A cut-off value of 1034.48 ng/µL was associated with a sensitivity of 68% and a specificity of 75%. The performance of an assay for the identification of BC subjects based on the AUC value of the ROC was 0.754 (bootstrapped AUC 0.749) a level commonly considered as acceptable/fair [33, 34].

The usefulness of the assay to consider a BC patient positive or negative for disease relapse at follow up should be better evaluated in further longitudinal studies. However, a correlation between EVs concentration and the presence or absence of tumor has been demonstrated in the present study since, for a subgroup of patients, it was possible to compare EVs plasmatic levels with the one present before the intervention. EVs levels post-surgery were lower than pre-surgery (*p* = 0.014) and not statistically different from HC. It must be noticed that, even if the median value of the pre-surgery levels of EVs of this subgroup of patients was slightly lower than those of the total cohort of BC, the two groups were not statistically different (Supplementary Fig. S6). On the contrary, pre-surgery levels are higher than HC (*p* = 0.00154).

Conversely, no correlations were found between EVs concentration and clinical characteristics of BC.

The dimension of EVs, extracted from a subgroup of subjects, was also studied. While the exact determination of EVs size is a complex task and no absolute technique is available [35], Nanosight analysis is currently considered as the gold standard as it allows to analyze the EVs directly in liquid [36]. The geometric means of the size in the different samples studied was constant around 120 nm and did not show a correlation with the levels of EVs measured by the assay in the untreated plasma both in BC and HC subjects. This lack of correlation excludes the possibility that the higher levels of CD9+/CD63+ observed in BC patients were due to the fractionation of the EVs in smaller particles. The most likely cause of the different plasmatic levels of EVs must then lie in the augmented production of EVs or by cancer cells, or by the organism as a response to the presence of the tumor. The geometric means was chosen as metric for the analysis as it is known that EVs are not only very polydisperse, but their dispersion is also non symmetric [37]. This situation is common to other particle distribution problems and the use of the geometric mean, instead of arithmetic mean, allows reducing the effect of the extreme data [38].

To detect circulating EVs in a cohort of early BC patients, outside a metastatic setting, could be a difficult challenge. Simpler and more accurate technologies are needed since the concentration of EVs in blood samples is generally low. The present study demonstrates the feasibility and the great advantage of EVs detection by SiMoA, as this approach does not require a prior purification of the target from plasma. In order to avoid the interference from circulating proteins, the assay is based on the detection of particles positive at both CD9 and CD63 tetraspanins. This choice limits the percentage of EVs detected from the pool of all EVs present in blood. In fact, it is well known that a rather high percentage of EVs does not present both the marker simultaneously [39]. On the other hand, this choice limits the interference by circulating fragments of the two proteins.

The apparent high value of EVs in term of ng/µL reported by the assay depends on the high molecular mass of extracellular vesicles. By converting, the mass of commercial standard used in the number of particles according to the indication provided by the supplier, the SiMoA assay was able to detect a number of particles in the range of 10^5^ for µL. However, it must be noted that for the calibration of the assay we used a commercial standard of particles extracted from human plasma. As such, only a small fraction of the number of particles present in the standard is effectively made of CD9 and CD63 positive extracellular vesicles, and a large number of other particles such as lipoproteins and protein aggregates are present in the standard but are not relevant for our assay. While we do not have exact data on the proportion of the contaminants the datasheet of the standards reports that about 30% of the particles present are CD9, CD63 or CD81 positive. As mentioned however, the SiMoA assay detects only particles that are simultaneously positive for CD9 and CD63, that are a further fraction of this 30%. According to this, the real limit of quantification is expected to be about 10^4^ EVs for microliter. This is also proved by the fact that we can measure EVs levels from very limited amount of plasma (4 µL).

The good performance of the SiMoA technology is due to the use of magnetic beads, conjugated with an antibody of interest, that are let in contact with the sample of interest to fish out the target protein or particle and with a biotinylated detection antibody. After the magnetic extraction, the system can dispose each microbead in a single femtoliter-sized well together with a streptavidin β-galactosidase and with a defined amount of substrate (RGP). By confining the fluorophores generated into an array of femtoliter-sized wells, SiMoA ensures a high local concentration of fluorescent signal even when very low concentrations of the target are present in the sample. As such, by using a standard microscopic optics to acquire fluorescence images of each well SiMoA can detect signals close to a single particle. As SiMoA is based on the use of magnetic beads, it can be considered also as an ELISA-like assay combined with an immunoseparation in a single run.

Combining these two steps in a single assay benefit the assay's reproducibility and scalability. The immunoseparation of EVs is considered a powerful approach that provides pure EVs easier than differential ultracentrifugation and was proposed earlier [40]. However, in many cases, a pre-purification step of EVs from blood plasma was required to decrease the proteins present that other ways would stick to the surface of the beads or of the solid phase reducing the reproducibility of the results [41, 42]. Thanks to the excellent detection limit of the SiMoA, this problem is reduced. In our assay, minimal amounts of plasma are diluted in a much larger volume of buffer, thus diluting the adhesion of free proteins on the surface of the beads.

Recently, other groups reported the direct extraction of EVs from whole plasma using magnetic beads with good results. However, their analytical methods usually require the release of the EVs from the surface of the beads by harsh conditions [43] or by using enzymatic steps [44] that introduce complexity in the workflow, thus affecting the clinical translation of the work.

The integrated assay here developed allowed us to measure 26 samples prepared in triplicate in each run, starting from an extremely limited amount of plasma and requires a total time from 4 to 5 h to be completed. This represents a much higher throughput if compared with the other techniques traditionally used for this task, and open to the possibility to conduct the large clinical trial needed to validate the use of EVs as a biomarker.

In the future, SiMoA could allow another great advantage: the possibility to test multiple EVs markers simultaneously. By using magnetic beads encoded with different colors SiMoA is able to measure up to four markers in a single run. This could allow measuring multiple subpopulations of EVs presenting markers more specific for cancer-related EVs (such as CD24, CD44, or CD340) [45]. As such, the use of SiMoA assays could improve the specificity, since the co-presence of at least two biomarkers is needed in the same EV to be correctly recognized and labeled by SiMoA. Therefore, false-positive cases due to separate detection of circulating free receptors would be greatly decreased.

Our study has some limitations. Indeed, we did not here test the overall diagnostic performance of SiMoA in making a definite diagnosis of BC, because this needs an external validation in an unselected population. However, this was far beyond our aim. Additionally, the monocentric nature of the study and the relatively small sample size might have mitigated some important differences in the levels of the EVs in relation to the tumor size.

In addition, the AUC value of the ROC curve here determined as 0.75 is lower than the one reported for other markers whose presence on the surface of EVs seems more specifically associated with BC [11, 16, 19]. In the near future, the combination of the clinical translatability of the SiMoA assay with the selection of more specific markers could deliver an accurate and easy to perform test of clinical utility for the management of BC based on the detection of EVs.

## Conclusion

SiMoA assay for the quantification of CD9+/CD63+ EVs allowed to drastically improve the study of the plasmatic levels of EVs directly from plasma without requiring any prior sample processing. EVs levels are significantly higher in BC patients if compared to those of HC, and EVs concentration significantly decreases after cancer surgery to reach levels equal to the one of the HC. Further studies will be planned in the next future to assess the role of EVs concentration in different stages of BC, as well as its role in predicting response to treatments in both adjuvant and neoadjuvant settings. Furthermore, more specific biomarkers of BC-related EVs will be discovered and assessed for a future implementation in clinical practice to personalize the therapeutic approach.

## Supplementary Information

Below is the link to the electronic supplementary material.

Supplementary file1 (DOCX 887 KB)

## Data Availability

The authors will provide data upon request.

## Abbreviations

EVs: Extracellular vesicles
BC: Breast cancer
HC: Healthy controls
SiMoA: Single Molecule Array
EDC: 1-Ethyl-3-(3-dimethylaminopropyl) carbodiimide hydrochloride
SBG: Streptavidin-β-galactosidase
RGP: Resorufin β-d-galactopyranoside
NTA: Nanoparticle-tracking analysis
ICH: International conference of harmonization
GCP: Good clinical practice
AEB: Average enzyme activity

## Code availability

Data analysis was performed using OriginLab, SAS (v. 9.4, SAS Institute Inc., Cary, USA) and R software (v. 3.5.1, © The R Foundation).
