# Supplementary material for: Fast quantification of extracellular vesicles levels in early breast cancer patients by Single Molecule Detection Array (SiMoA)
Source: Breast Cancer Res Treat. 2021 Dec 21;192(1):65–74. doi: 10.1007/s10549-021-06474-3 (PMC8841315; doi:10.1007/s10549-021-06474-3)
Supplement: Supplementary file 1 — Supplementary file1 (DOCX 887 KB) [file 10549_2021_6474_MOESM1_ESM.docx]

**Supplementary information**

# Fast quantification of extracellular vesicles levels in early breast cancer patients by Single Molecule Detection Assay (SiMoA).

Carlo Morasso^1#^, Alessandra Ricciardi^1#^, Daisy Sproviero^2^, Marta Truffi^1^, Sara Albasini^3^, Francesca Piccotti^1^, Federico Sottotetti^4^, Ludovica Mollica^4^, Cristina Cereda^2^, Luca Sorrentino^5^, Fabio Corsi^3,6*^

1. Laboratory of Nanomedicine, Istituti Clinici Scientifici Maugeri IRCCS, Pavia, Italy.
2. Genomic and post-Genomic Center, IRCCS Mondino Foundation, Pavia, Italy.
3. Breast Unit, Istituti Clinici Scientifici Maugeri IRCCS, Pavia, Italy.
4. Medical Oncology Unit, Istituti Clinici Scientifici Maugeri IRCCS, Pavia, Italy.
5. Colorectal Surgery Unit, Fondazione IRCCS Istituto Nazionale dei Tumori di Milano, Milan, Italy.
6. Dipartimento di Scienze Biomediche e Cliniche “L. Sacco”, Università di Milano, 20157 Milano, Italy.

**Supplementary Tables**

**Table S1.** Correlation between EVs concentration and age or BMI.

| **Spearman correlation and relative p-value** | | | |
| --- | --- | --- | --- |
|  | | **Age** | **BMI** |
| **HC** | **EVs’ level (ng/µl)** | - 0.02  p = 0.84 | 0.04  p = 0.75 |
| **BC** | **EVs’ level (ng/µl)** | 0.08  p = 0.41 | - 0.09  p = 0.46 |

**Table S2.** Correlation between EVs concentration and clinical features.

|  | **Median EVs concentration [Q1 - Q3]** | **P Value** |
| --- | --- | --- |
| **pT** |  |  |
| 1 | 1643.1 [536.5 – 4094.3] | 0.07* |
| 2 | 3011.7 [1238.7 – 5645.2] |  |
| **pN** |  |  |
| 0 | 1703.2 [540.7 – 3681.8] | 0.23* |
| 1 | 2587.9 [1102.8 – 5895.4] |  |
| **Grading** |  |  |
| 1 | 3169.6 [1218.5 – 10605.3] | 0.15** |
| 2 | 1539.8 [622.0 – 3729.5] |  |
| 3 | 2328.3 [551.3 – 5087.9] |  |
| **Molecular subtype** |  |  |
| Luminal A | 1729.3 [720.2 - 4569.7] | 0.53** |
| Luminal B | 2752.1 [846.7 – 5645.2] |  |
| Triple-negative | 1200.6 [413.8 – 2310.1] |  |

* p-value from Mann-Whitney Test

** p-value from Kruskal-Wallis Test

**Supplementary Figures**





**Fig S1.** ROC Curve relative to the classification of BC and HC subjects based on the plasmatic levels of EVs

**Fig S2.** Correlation between the geometric mean of the whole population of EVs extracted from the plasma sample of a subgroup of subjects (TOT n=16; BC n=10; HC n=6) and the plasmatic levels of CD9+ / CD63+ EVs measured by the SiMoA assay.


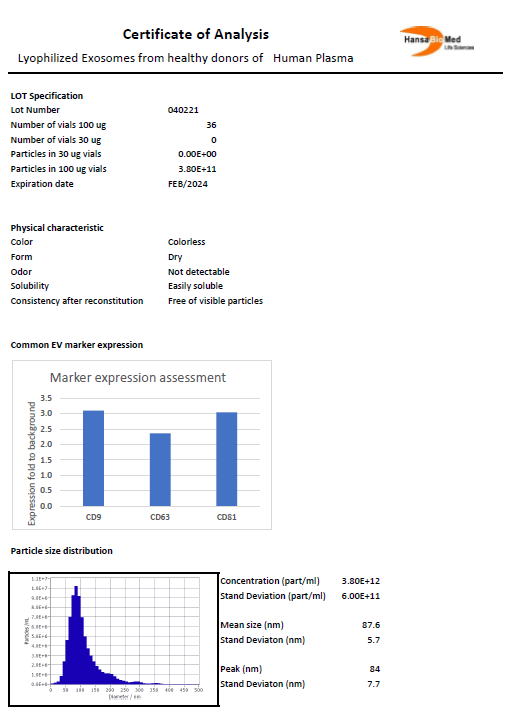


**Fig S3**. Certificate of analysis of the EVs standard used in the assay (data provided by the supplier).


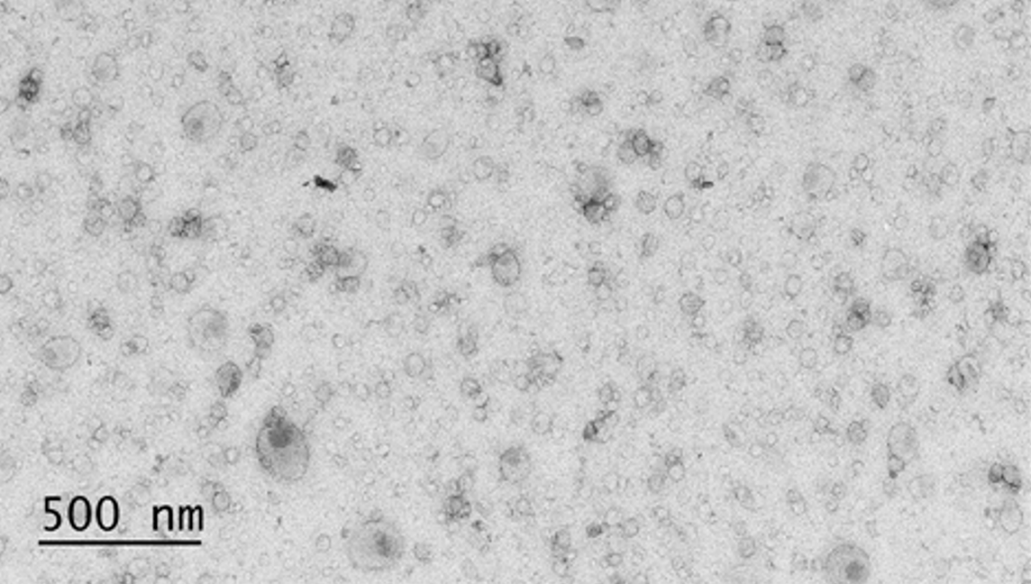


**FigS4**. TEM image of the EVs used as standard (data provided by the supplier).


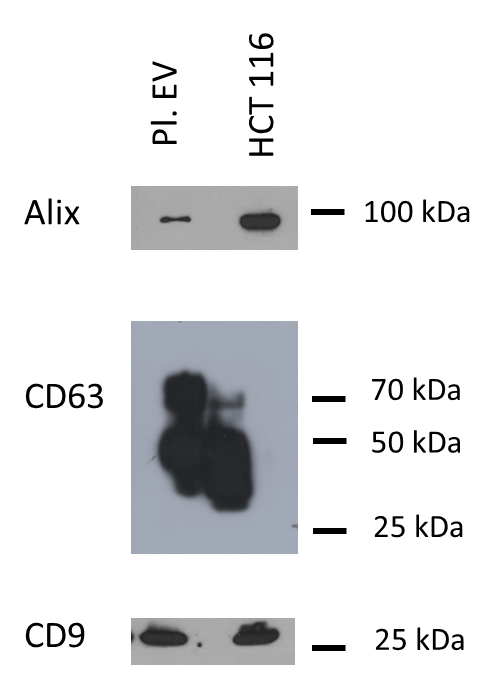


**FigS5**. Western blot of commonly employed markers for EVs of the standard used in the SiMoA assay (data provided by the supplier).





**FigS6**. Plasmatic EVs levels measured in all the BC included in the study (n=95) and in subgroup of patients for witch EVs levels one month after surgery are available (n=45). Data are shown as box and whisker plots. Each data point represents an individual subject analysed. Each box represents the area between the 25th and 75th percentiles [interquartile range, IQR]. Lines inside the boxes represent the median values. White dots represent the mean value for each class. Whiskers extend to the lowest and highest values within 1.5 times the IQR from the box.
